# [5,15-Bis(2-methyl­prop­yl)porphyrinato]nickel(II)

**DOI:** 10.1107/S1600536812035726

**Published:** 2012-08-23

**Authors:** Mathias O. Senge

**Affiliations:** a School of Chemistry, SFI Tetrapyrrole Laboratory, Trinity Biomedical Sciences Institute, 152-160 Pearse Street, Trinity College Dublin, Dublin 2, Ireland

## Abstract

The title compound, [Ni(C_28_H_28_N_4_)], crystallizes with two independent mol­ecules in the unit cell, one of which is located on an inversion center. Both macrocycles exhibit a planar conformation with average deviation from the least-squares-plane of the 24 macrocycle atoms of Δ24 = 0.043 Å for the first mol­ecule and 0.026 Å for the mol­ecule located on an inversion center. The average Ni—N bond lengths are 1.955 (2) and 1.956 (2) Å in the two mol­ecules. The mol­ecules form π–π dimers of inter­mediary strength with a mean plane separation of 3.36 (2) Å.

## Related literature

For the conformation of porphyrins, see: Senge (2006 ▶). For the structural analysis of π-aggregates, see: Scheidt & Lee (1987 ▶). For Ni(II) porphyrin structures, see: Song *et al.* (1996 ▶, 1998 ▶); Davis *et al.* (2010 ▶); Jentzen *et al.* (1996 ▶); Senge & Davis (2010 ▶); Senge *et al.* (2000 ▶, 2010 ▶);. For the preparation, see: Wiehe *et al.* (2005 ▶).
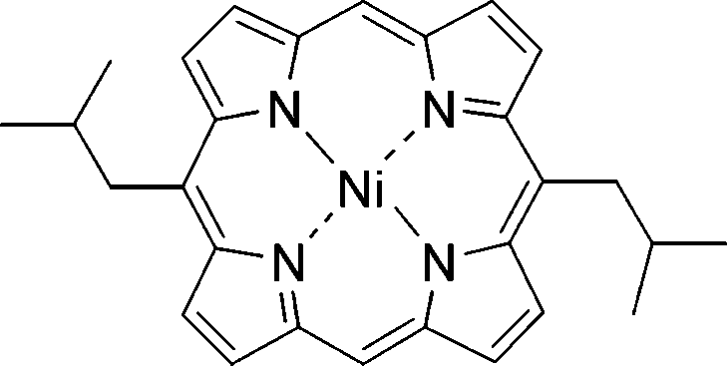


## Experimental

### 

#### Crystal data

[Ni(C_28_H_28_N_4_)]
*M* *_r_* = 479.25
Triclinic, 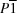

*a* = 9.951 (2) Å
*b* = 13.197 (3) Å
*c* = 13.700 (3) Å
α = 73.03 (3)°
β = 75.27 (3)°
γ = 73.39 (3)°
*V* = 1620.1 (7) Å^3^
*Z* = 3
Mo *K*α radiation
μ = 0.92 mm^−1^
*T* = 90 K
0.20 × 0.15 × 0.01 mm

#### Data collection

Bruker SMART APEXII diffractometer
Absorption correction: multi-scan (*SADABS*; Bruker, 2005 ▶) *T*_min_ = 0.837, *T*_max_ = 0.995
21454 measured reflections
7431 independent reflections
5881 reflections with *I* > 2σ(*I*)
*R*_int_ = 0.033

#### Refinement

*R*[*F* ^2^ > 2σ(*F* ^2^)] = 0.033
*wR*(*F* ^2^) = 0.096
*S* = 1.04
7431 reflections
454 parameters
H-atom parameters constrained
Δρ_max_ = 0.73 e Å^−3^
Δρ_min_ = −0.38 e Å^−3^

### 

Data collection: *APEX2* (Bruker, 2005 ▶); cell refinement: *SAINT* (Bruker, 2005 ▶); data reduction: *SAINT*; program(s) used to solve structure: *SHELXS97* (Sheldrick, 2008 ▶); program(s) used to refine structure: *SHELXL97* (Sheldrick, 2008 ▶); molecular graphics: *XP* in *SHELXTL* (Sheldrick, 2008 ▶); software used to prepare material for publication: *SHELXTL*.

## Supplementary Material

Crystal structure: contains datablock(s) I, global. DOI: 10.1107/S1600536812035726/rn2107sup1.cif

Structure factors: contains datablock(s) I. DOI: 10.1107/S1600536812035726/rn2107Isup2.hkl

Additional supplementary materials:  crystallographic information; 3D view; checkCIF report

## Figures and Tables

**Table 1:** Selected bond lengths (Å)

Ni1—N24	1.9497 (16)
Ni1—N22	1.9508 (16)
Ni1—N23	1.9595 (15)
Ni1—N21	1.9623 (15)
Ni2—N28	1.9537 (16)
Ni2—N25	1.9580 (15)

**Table 2:** Comparison of Ni^II^ 5,15-dialkyl­porphyrins (Å, °) Δ is the deviation from the least-squares-plane of the 24 macrocycle atoms and N—Ni—Nadj is the angle between neighboring pyrrole units.

Alkyl residue	*tert*-But­yl	Isoprop­yl	*iso*-But­yl	none
Ni—N	1.897 (2)	1.930 (2)	1.955 (2)	1.951 (2)
Δ	0.4	0.26	0.04	0.02
N—Ni—Nadj	92.3, 87.7	91.8, 88.2	91.6, 88.4	90, 90
Reference	Song *et al.* (1996 ▶)	Song *et al.* (1998 ▶)	This work	Jentzen *et al.* (1996 ▶)

**Fig. 1.**
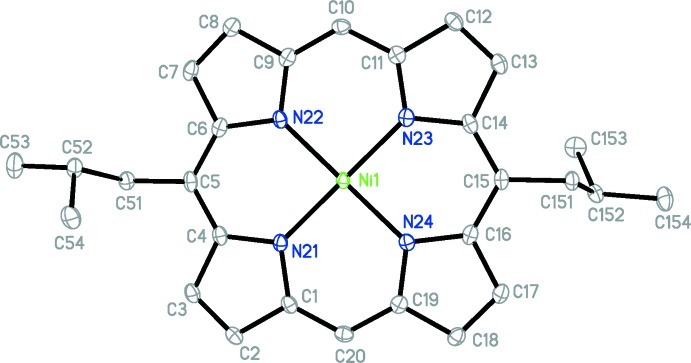
: Molecular structure of molecule 1 of the title compound. Thermal ellipsoids are drawn at 50% probability level; hydrogen atoms have been omitted for clarity.

**Fig. 2.**
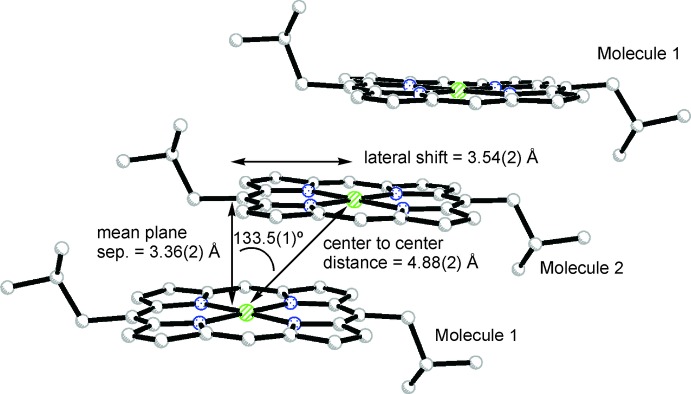
: View of the π-aggregates formed by the title compound in the crystal.

## supplementary crystallographic information

### Comment

*meso*-Alkylporphyrins are increasingly used in porphyrin chemistry, but their structural chemistry is less well established (Senge *et al.*, 2010). The compound is another example for the expanding body of Ni(II) porphyrins with a planar macrocycle (Davis *et al.*, 2010; Jentzen *et al.*, 1996; Senge & Davis (2010). In the crystal this allows the formation of π-aggregates which are characterized by a mean plane separation of 3.36 (2) Å, a center-to-center distance of 4.88 (2) Å, a slip angle of 133.5 (1) ° which, according to the classification given by Scheidt & Lee (1987), results in a lateral shift of the metal centers of 3.54 (2) Å. Thus, the π-π-stacks are of intermediary strength. The compound forms part of a series of Ni(II) 5,15-dialkylporphyrins with different steric demand of the *meso* residue. The respective *tert*-butyl derivative (Song *et al.*, 1996) is clearly the most nonplanar one with the shortest Ni—N bond length and largest deviation from planarity (Table 2). The *iso*-propyl derivative (Song *et al.*, 1998) shows still significant out-of-plane deformations, while Ni(II)porphyrin without any non-hydrogen residues is planar (Jentzen *et al.*, 1996). The title compound has the sterically least demanding alkyl residue and exhibits an almost planar macrocycle. However, as indicated by the different N—Ni—N adj bond angles, the compound still exhibits some degree of in-plane distortion, which becomes more pronounced with larger *meso* alkyl residues.

### Experimental

The compound was prepared as described by Wiehe *et al.* (2005) and crystallized from CH_2_Cl_2_/CH_3_OH.

### Refinement

All nonhydrogen atoms were refined with anisotropic thermal parameters. Hydrogen atoms were refined with a standard riding model (C—H distance 0.96 Å, *U*_iso_ = 0.05).

### Crystal data

**Table:**

[Ni(C_28_H_28_N_4_)]	*Z* = 3
*M**_r_* = 479.25	*F*(000) = 756
Triclinic, *P*1	*D*_x_ = 1.474 Mg m^−^^3^*D*_m_ = n/d Mg m^−^^3^*D*_m_ measured by not measured
Hall symbol: -P 1	Melting point: n/d K
*a* = 9.951 (2) Å	Mo *K*α radiation, λ = 0.71073 Å
*b* = 13.197 (3) Å	Cell parameters from 7215 reflections
*c* = 13.700 (3) Å	θ = 4.8–62.9°
α = 73.03 (3)°	µ = 0.92 mm^−^^1^
β = 75.27 (3)°	*T* = 90 K
γ = 73.39 (3)°	Plate, red
*V* = 1620.1 (7) Å^3^	0.20 × 0.15 × 0.01 mm

### Data collection

**Table:**

Bruker SMART APEXII diffractometer	7431 independent reflections
Radiation source: fine-focus sealed tube	5881 reflections with *I* > 2σ(*I*)
Graphite monochromator	*R*_int_ = 0.033
Detector resolution: 8.3 pixels mm^-1^	θ_max_ = 27.6°, θ_min_ = 2.0°
ω scans	*h* = −12→12
Absorption correction: multi-scan (*SADABS*; Bruker, 2005)	*k* = −17→17
*T*_min_ = 0.837, *T*_max_ = 0.995	*l* = −17→17
21454 measured reflections	

### Refinement

**Table:**

Refinement on *F*^2^	Primary atom site location: heavy-atom method
Least-squares matrix: full	Secondary atom site location: difference Fourier map
*R*[*F*^2^ > 2σ(*F*^2^)] = 0.033	Hydrogen site location: inferred from neighbouring sites
*wR*(*F*^2^) = 0.096	H-atom parameters constrained
*S* = 1.04	*w* = 1/[σ^2^(*F*_o_^2^) + (0.0528*P*)^2^ + 0.238*P*] where *P* = (*F*_o_^2^ + 2*F*_c_^2^)/3
7431 reflections	(Δ/σ)_max_ = 0.001
454 parameters	Δρ_max_ = 0.73 e Å^−^^3^
0 restraints	Δρ_min_ = −0.38 e Å^−^^3^

### Special details

**Table:**

Geometry. All e.s.d.'s (except the e.s.d. in the dihedral angle between two l.s. planes) are estimated using the full covariance matrix. The cell e.s.d.'s are taken into account individually in the estimation of e.s.d.'s in distances, angles and torsion angles; correlations between e.s.d.'s in cell parameters are only used when they are defined by crystal symmetry. An approximate (isotropic) treatment of cell e.s.d.'s is used for estimating e.s.d.'s involving l.s. planes.
Refinement. Refinement of *F*^2^ against ALL reflections. The weighted *R*-factor *wR* and goodness of fit *S* are based on *F*^2^, conventional *R*-factors *R* are based on *F*, with *F* set to zero for negative *F*^2^. The threshold expression of *F*^2^ > σ(*F*^2^) is used only for calculating *R*-factors(gt) *etc*. and is not relevant to the choice of reflections for refinement. *R*-factors based on *F*^2^ are statistically about twice as large as those based on *F*, and *R*- factors based on ALL data will be even larger.

### Fractional atomic coordinates and isotropic or equivalent isotropic displacement parameters (Å^2^)

**Table:**

	*x*	*y*	*z*	*U*_iso_*/*U*_eq_	
Ni1	0.01600 (2)	0.178583 (17)	0.287430 (16)	0.01052 (7)	
N21	0.09964 (15)	0.13788 (11)	0.41252 (11)	0.0120 (3)	
N22	−0.17178 (15)	0.18586 (11)	0.37693 (11)	0.0119 (3)	
N23	−0.06732 (15)	0.21932 (11)	0.16241 (11)	0.0123 (3)	
N24	0.20411 (15)	0.16763 (11)	0.19834 (11)	0.0117 (3)	
C1	0.24215 (19)	0.11741 (14)	0.41507 (13)	0.0133 (3)	
C2	0.26587 (19)	0.08745 (14)	0.51990 (13)	0.0151 (4)	
H2A	0.3549	0.0709	0.5412	0.018*	
C3	0.13722 (19)	0.08738 (14)	0.58197 (14)	0.0152 (4)	
H3A	0.1186	0.0696	0.6558	0.018*	
C4	0.03290 (19)	0.11912 (13)	0.51629 (13)	0.0132 (3)	
C5	−0.11240 (19)	0.12848 (13)	0.55287 (13)	0.0135 (3)	
C6	−0.20684 (18)	0.16178 (13)	0.48477 (13)	0.0134 (3)	
C7	−0.35888 (19)	0.17765 (14)	0.51781 (14)	0.0156 (4)	
H7A	−0.4091	0.1656	0.5876	0.019*	
C8	−0.41722 (19)	0.21270 (14)	0.43196 (14)	0.0152 (4)	
H8A	−0.5160	0.2313	0.4294	0.018*	
C9	−0.30068 (19)	0.21646 (14)	0.34503 (14)	0.0137 (3)	
C10	−0.32030 (19)	0.24546 (14)	0.24399 (13)	0.0143 (3)	
H10A	−0.4154	0.2667	0.2318	0.017*	
C11	−0.21063 (19)	0.24565 (13)	0.15931 (13)	0.0133 (3)	
C12	−0.23387 (19)	0.27354 (14)	0.05493 (13)	0.0152 (4)	
H12A	−0.3234	0.2946	0.0334	0.018*	
C13	−0.10439 (19)	0.26420 (14)	−0.00671 (14)	0.0154 (4)	
H13A	−0.0853	0.2775	−0.0803	0.019*	
C14	−0.00007 (19)	0.23047 (13)	0.05900 (13)	0.0126 (3)	
C15	0.14609 (19)	0.21209 (13)	0.02340 (13)	0.0128 (3)	
C16	0.24007 (18)	0.18209 (13)	0.09133 (13)	0.0134 (3)	
C17	0.39244 (19)	0.16363 (14)	0.05829 (14)	0.0153 (4)	
H17A	0.4433	0.1683	−0.0111	0.018*	
C18	0.44959 (19)	0.13858 (14)	0.14356 (13)	0.0150 (4)	
H18A	0.5482	0.1223	0.1462	0.018*	
C19	0.33268 (18)	0.14126 (13)	0.22971 (13)	0.0132 (3)	
C20	0.35183 (19)	0.11896 (14)	0.33050 (14)	0.0144 (3)	
H20A	0.4464	0.1038	0.3423	0.017*	
C51	−0.17085 (19)	0.10162 (14)	0.66844 (13)	0.0144 (3)	
H51A	−0.0921	0.0545	0.7042	0.017*	
H51B	−0.2432	0.0588	0.6813	0.017*	
C52	−0.23936 (19)	0.19972 (14)	0.71841 (13)	0.0163 (4)	
H52A	−0.3204	0.2464	0.6834	0.020*	
C53	−0.2991 (2)	0.15754 (17)	0.83269 (14)	0.0247 (4)	
H53A	−0.3481	0.2192	0.8644	0.037*	
H53B	−0.2208	0.1125	0.8685	0.037*	
H53C	−0.3668	0.1138	0.8387	0.037*	
C54	−0.1336 (2)	0.26873 (14)	0.70584 (14)	0.0192 (4)	
H54A	−0.1823	0.3318	0.7356	0.029*	
H54B	−0.0956	0.2938	0.6319	0.029*	
H54C	−0.0551	0.2249	0.7419	0.029*	
C151	0.20745 (19)	0.22250 (14)	−0.09139 (13)	0.0144 (4)	
H15A	0.2840	0.2628	−0.1108	0.017*	
H15B	0.1315	0.2664	−0.1313	0.017*	
C152	0.26898 (19)	0.11315 (14)	−0.12369 (13)	0.0156 (4)	
H15C	0.3450	0.0692	−0.0826	0.019*	
C153	0.1561 (2)	0.04767 (15)	−0.10150 (15)	0.0211 (4)	
H15D	0.1999	−0.0209	−0.1229	0.032*	
H15E	0.0798	0.0897	−0.1403	0.032*	
H15F	0.1162	0.0322	−0.0270	0.032*	
C154	0.3382 (2)	0.13526 (15)	−0.23833 (14)	0.0215 (4)	
H15G	0.3870	0.0661	−0.2575	0.032*	
H15H	0.4074	0.1797	−0.2508	0.032*	
H15I	0.2645	0.1742	−0.2804	0.032*	
Ni2	0.0000	0.5000	0.0000	0.01010 (8)	
N25	0.07725 (16)	0.46002 (11)	0.12751 (11)	0.0121 (3)	
N28	0.19117 (15)	0.48543 (11)	−0.08547 (11)	0.0119 (3)	
C21	0.21889 (19)	0.43651 (13)	0.13368 (13)	0.0132 (3)	
C22	0.2379 (2)	0.41090 (14)	0.23900 (14)	0.0160 (4)	
H22A	0.3258	0.3936	0.2622	0.019*	
C23	0.10709 (19)	0.41616 (14)	0.29854 (14)	0.0158 (4)	
H23A	0.0852	0.4020	0.3722	0.019*	
C24	0.00630 (19)	0.44711 (13)	0.23025 (13)	0.0130 (3)	
C25	−0.14021 (19)	0.46298 (13)	0.26333 (13)	0.0127 (3)	
C36	0.23108 (18)	0.50284 (13)	−0.19230 (13)	0.0126 (3)	
C37	0.38420 (19)	0.48051 (14)	−0.22192 (14)	0.0155 (4)	
H37A	0.4376	0.4863	−0.2907	0.019*	
C38	0.43770 (19)	0.44991 (14)	−0.13422 (14)	0.0155 (4)	
H38A	0.5357	0.4293	−0.1290	0.019*	
C39	0.31770 (18)	0.45454 (13)	−0.05027 (13)	0.0132 (3)	
C40	0.33182 (19)	0.43296 (14)	0.05146 (14)	0.0144 (3)	
H40A	0.4255	0.4144	0.0659	0.017*	
C251	−0.20483 (19)	0.44345 (14)	0.37852 (13)	0.0143 (4)	
H25B	−0.1304	0.3949	0.4178	0.017*	
H25C	−0.2818	0.4047	0.3918	0.017*	
C252	−0.26687 (19)	0.54652 (14)	0.42135 (13)	0.0147 (4)	
H25D	−0.3437	0.5947	0.3827	0.018*	
C253	−0.3340 (2)	0.51309 (15)	0.53581 (14)	0.0217 (4)	
H25E	−0.3883	0.5782	0.5608	0.033*	
H25F	−0.2587	0.4737	0.5763	0.033*	
H25G	−0.3982	0.4659	0.5437	0.033*	
C254	−0.1548 (2)	0.61033 (15)	0.40823 (15)	0.0197 (4)	
H25H	−0.1994	0.6750	0.4362	0.029*	
H25I	−0.1145	0.6327	0.3343	0.029*	
H25J	−0.0787	0.5643	0.4457	0.029*	

### Atomic displacement parameters (Å^2^)

**Table:**

	*U*^11^	*U*^22^	*U*^33^	*U*^12^	*U*^13^	*U*^23^
Ni1	0.01265 (12)	0.01100 (12)	0.00933 (12)	−0.00463 (9)	−0.00089 (8)	−0.00364 (8)
N21	0.0146 (7)	0.0121 (7)	0.0108 (7)	−0.0055 (6)	−0.0006 (6)	−0.0039 (6)
N22	0.0159 (7)	0.0116 (7)	0.0100 (7)	−0.0059 (6)	−0.0018 (6)	−0.0032 (5)
N23	0.0152 (7)	0.0104 (7)	0.0124 (7)	−0.0041 (6)	−0.0014 (6)	−0.0042 (6)
N24	0.0146 (7)	0.0104 (7)	0.0116 (7)	−0.0049 (6)	−0.0025 (6)	−0.0031 (5)
C1	0.0176 (9)	0.0119 (8)	0.0127 (8)	−0.0053 (7)	−0.0038 (7)	−0.0035 (7)
C2	0.0181 (9)	0.0150 (8)	0.0144 (9)	−0.0036 (7)	−0.0061 (7)	−0.0045 (7)
C3	0.0202 (9)	0.0156 (8)	0.0126 (8)	−0.0060 (7)	−0.0034 (7)	−0.0053 (7)
C4	0.0193 (9)	0.0106 (8)	0.0113 (8)	−0.0051 (7)	−0.0022 (7)	−0.0036 (6)
C5	0.0214 (9)	0.0102 (8)	0.0111 (8)	−0.0062 (7)	−0.0008 (7)	−0.0050 (6)
C6	0.0162 (9)	0.0109 (8)	0.0144 (8)	−0.0068 (7)	0.0017 (7)	−0.0054 (7)
C7	0.0168 (9)	0.0170 (9)	0.0151 (9)	−0.0081 (7)	0.0030 (7)	−0.0080 (7)
C8	0.0151 (9)	0.0149 (8)	0.0178 (9)	−0.0061 (7)	−0.0002 (7)	−0.0069 (7)
C9	0.0153 (9)	0.0118 (8)	0.0165 (9)	−0.0066 (7)	0.0002 (7)	−0.0065 (7)
C10	0.0126 (8)	0.0147 (8)	0.0180 (9)	−0.0036 (7)	−0.0044 (7)	−0.0058 (7)
C11	0.0167 (9)	0.0100 (8)	0.0148 (9)	−0.0039 (7)	−0.0034 (7)	−0.0040 (7)
C12	0.0185 (9)	0.0140 (8)	0.0151 (9)	−0.0035 (7)	−0.0058 (7)	−0.0041 (7)
C13	0.0207 (9)	0.0143 (8)	0.0125 (8)	−0.0040 (7)	−0.0040 (7)	−0.0042 (7)
C14	0.0197 (9)	0.0087 (8)	0.0111 (8)	−0.0038 (7)	−0.0043 (7)	−0.0033 (6)
C15	0.0197 (9)	0.0095 (8)	0.0098 (8)	−0.0056 (7)	−0.0003 (7)	−0.0029 (6)
C16	0.0164 (9)	0.0114 (8)	0.0132 (8)	−0.0058 (7)	0.0012 (7)	−0.0049 (7)
C17	0.0164 (9)	0.0157 (8)	0.0142 (9)	−0.0058 (7)	0.0012 (7)	−0.0056 (7)
C18	0.0147 (9)	0.0149 (8)	0.0163 (9)	−0.0058 (7)	−0.0002 (7)	−0.0048 (7)
C19	0.0153 (9)	0.0107 (8)	0.0153 (9)	−0.0056 (7)	−0.0010 (7)	−0.0047 (7)
C20	0.0147 (9)	0.0135 (8)	0.0175 (9)	−0.0045 (7)	−0.0047 (7)	−0.0048 (7)
C51	0.0168 (9)	0.0146 (8)	0.0126 (8)	−0.0073 (7)	−0.0005 (7)	−0.0029 (7)
C52	0.0180 (9)	0.0174 (9)	0.0140 (9)	−0.0051 (7)	−0.0007 (7)	−0.0057 (7)
C53	0.0296 (11)	0.0291 (11)	0.0170 (10)	−0.0108 (9)	0.0026 (8)	−0.0098 (8)
C54	0.0256 (10)	0.0170 (9)	0.0193 (9)	−0.0083 (8)	−0.0046 (8)	−0.0069 (7)
C151	0.0166 (9)	0.0166 (9)	0.0101 (8)	−0.0046 (7)	−0.0009 (7)	−0.0040 (7)
C152	0.0194 (9)	0.0153 (8)	0.0123 (8)	−0.0030 (7)	−0.0044 (7)	−0.0036 (7)
C153	0.0242 (10)	0.0162 (9)	0.0254 (10)	−0.0048 (8)	−0.0051 (8)	−0.0080 (8)
C154	0.0283 (10)	0.0212 (10)	0.0138 (9)	−0.0027 (8)	−0.0012 (8)	−0.0074 (7)
Ni2	0.01232 (16)	0.01096 (15)	0.00837 (15)	−0.00425 (12)	−0.00116 (11)	−0.00341 (11)
N25	0.0144 (7)	0.0115 (7)	0.0115 (7)	−0.0048 (6)	−0.0012 (6)	−0.0038 (6)
N28	0.0149 (7)	0.0113 (7)	0.0115 (7)	−0.0049 (6)	−0.0021 (6)	−0.0041 (6)
C21	0.0177 (9)	0.0100 (8)	0.0138 (8)	−0.0040 (7)	−0.0048 (7)	−0.0032 (7)
C22	0.0183 (9)	0.0166 (9)	0.0151 (9)	−0.0029 (7)	−0.0053 (7)	−0.0062 (7)
C23	0.0198 (9)	0.0159 (9)	0.0126 (8)	−0.0035 (7)	−0.0036 (7)	−0.0047 (7)
C24	0.0199 (9)	0.0090 (8)	0.0116 (8)	−0.0048 (7)	−0.0027 (7)	−0.0035 (6)
C25	0.0204 (9)	0.0095 (8)	0.0102 (8)	−0.0062 (7)	−0.0007 (7)	−0.0042 (6)
C36	0.0160 (9)	0.0111 (8)	0.0120 (8)	−0.0063 (7)	0.0015 (7)	−0.0048 (6)
C37	0.0175 (9)	0.0174 (9)	0.0135 (8)	−0.0078 (7)	0.0013 (7)	−0.0066 (7)
C38	0.0141 (9)	0.0161 (9)	0.0173 (9)	−0.0054 (7)	0.0005 (7)	−0.0065 (7)
C39	0.0148 (8)	0.0110 (8)	0.0148 (9)	−0.0052 (7)	−0.0004 (7)	−0.0045 (7)
C40	0.0129 (8)	0.0140 (8)	0.0182 (9)	−0.0033 (7)	−0.0048 (7)	−0.0047 (7)
C251	0.0198 (9)	0.0132 (8)	0.0108 (8)	−0.0067 (7)	−0.0005 (7)	−0.0035 (7)
C252	0.0188 (9)	0.0133 (8)	0.0120 (8)	−0.0029 (7)	−0.0024 (7)	−0.0041 (7)
C253	0.0278 (10)	0.0216 (10)	0.0141 (9)	−0.0044 (8)	0.0004 (8)	−0.0068 (7)
C254	0.0254 (10)	0.0163 (9)	0.0207 (9)	−0.0064 (8)	−0.0054 (8)	−0.0070 (7)

### Geometric parameters (Å, º)

**Table:**

Ni1—N24	1.9497 (16)	C54—H54A	0.9800
Ni1—N22	1.9508 (16)	C54—H54B	0.9800
Ni1—N23	1.9595 (15)	C54—H54C	0.9800
Ni1—N21	1.9623 (15)	C151—C152	1.544 (2)
N21—C1	1.373 (2)	C151—H15A	0.9900
N21—C4	1.390 (2)	C151—H15B	0.9900
N22—C9	1.373 (2)	C152—C154	1.524 (2)
N22—C6	1.390 (2)	C152—C153	1.526 (3)
N23—C11	1.377 (2)	C152—H15C	1.0000
N23—C14	1.389 (2)	C153—H15D	0.9800
N24—C19	1.371 (2)	C153—H15E	0.9800
N24—C16	1.387 (2)	C153—H15F	0.9800
C1—C20	1.375 (2)	C154—H15G	0.9800
C1—C2	1.436 (2)	C154—H15H	0.9800
C2—C3	1.345 (3)	C154—H15I	0.9800
C2—H2A	0.9500	Ni2—N28^i^	1.9537 (16)
C3—C4	1.438 (2)	Ni2—N28	1.9537 (16)
C3—H3A	0.9500	Ni2—N25^i^	1.9580 (15)
C4—C5	1.387 (3)	Ni2—N25	1.9580 (15)
C5—C6	1.385 (2)	N25—C21	1.373 (2)
C5—C51	1.514 (2)	N25—C24	1.390 (2)
C6—C7	1.437 (2)	N28—C39	1.373 (2)
C7—C8	1.346 (3)	N28—C36	1.383 (2)
C7—H7A	0.9500	C21—C40	1.375 (3)
C8—C9	1.436 (2)	C21—C22	1.432 (2)
C8—H8A	0.9500	C22—C23	1.344 (3)
C9—C10	1.373 (2)	C22—H22A	0.9500
C10—C11	1.376 (2)	C23—C24	1.438 (2)
C10—H10A	0.9500	C23—H23A	0.9500
C11—C12	1.430 (2)	C24—C25	1.385 (3)
C12—C13	1.344 (3)	C25—C36^i^	1.388 (2)
C12—H12A	0.9500	C25—C251	1.521 (2)
C13—C14	1.436 (2)	C36—C25^i^	1.388 (2)
C13—H13A	0.9500	C36—C37	1.439 (2)
C14—C15	1.385 (2)	C37—C38	1.345 (3)
C15—C16	1.383 (3)	C37—H37A	0.9500
C15—C151	1.516 (2)	C38—C39	1.433 (2)
C16—C17	1.438 (2)	C38—H38A	0.9500
C17—C18	1.343 (3)	C39—C40	1.374 (2)
C17—H17A	0.9500	C40—H40A	0.9500
C18—C19	1.432 (2)	C251—C252	1.542 (2)
C18—H18A	0.9500	C251—H25B	0.9900
C19—C20	1.376 (2)	C251—H25C	0.9900
C20—H20A	0.9500	C252—C253	1.527 (2)
C51—C52	1.544 (2)	C252—C254	1.527 (2)
C51—H51A	0.9900	C252—H25D	1.0000
C51—H51B	0.9900	C253—H25E	0.9800
C52—C53	1.524 (3)	C253—H25F	0.9800
C52—C54	1.526 (2)	C253—H25G	0.9800
C52—H52A	1.0000	C254—H25H	0.9800
C53—H53A	0.9800	C254—H25I	0.9800
C53—H53B	0.9800	C254—H25J	0.9800
C53—H53C	0.9800		
			
N24—Ni1—N22	178.64 (6)	C52—C54—H54A	109.5
N24—Ni1—N23	88.41 (6)	C52—C54—H54B	109.5
N22—Ni1—N23	91.64 (6)	H54A—C54—H54B	109.5
N24—Ni1—N21	91.53 (6)	C52—C54—H54C	109.5
N22—Ni1—N21	88.42 (6)	H54A—C54—H54C	109.5
N23—Ni1—N21	179.93 (7)	H54B—C54—H54C	109.5
C1—N21—C4	104.64 (14)	C15—C151—C152	114.61 (14)
C1—N21—Ni1	126.10 (12)	C15—C151—H15A	108.6
C4—N21—Ni1	129.22 (12)	C152—C151—H15A	108.6
C9—N22—C6	104.52 (14)	C15—C151—H15B	108.6
C9—N22—Ni1	126.38 (12)	C152—C151—H15B	108.6
C6—N22—Ni1	129.09 (12)	H15A—C151—H15B	107.6
C11—N23—C14	104.41 (14)	C154—C152—C153	111.25 (15)
C11—N23—Ni1	126.11 (12)	C154—C152—C151	108.95 (14)
C14—N23—Ni1	129.47 (12)	C153—C152—C151	112.31 (15)
C19—N24—C16	104.29 (14)	C154—C152—H15C	108.1
C19—N24—Ni1	126.62 (12)	C153—C152—H15C	108.1
C16—N24—Ni1	129.08 (12)	C151—C152—H15C	108.1
N21—C1—C20	125.99 (16)	C152—C153—H15D	109.5
N21—C1—C2	111.28 (16)	C152—C153—H15E	109.5
C20—C1—C2	122.66 (16)	H15D—C153—H15E	109.5
C3—C2—C1	106.52 (16)	C152—C153—H15F	109.5
C3—C2—H2A	126.7	H15D—C153—H15F	109.5
C1—C2—H2A	126.7	H15E—C153—H15F	109.5
C2—C3—C4	107.54 (15)	C152—C154—H15G	109.5
C2—C3—H3A	126.2	C152—C154—H15H	109.5
C4—C3—H3A	126.2	H15G—C154—H15H	109.5
C5—C4—N21	125.86 (16)	C152—C154—H15I	109.5
C5—C4—C3	124.13 (16)	H15G—C154—H15I	109.5
N21—C4—C3	110.01 (15)	H15H—C154—H15I	109.5
C6—C5—C4	120.85 (16)	N28^i^—Ni2—N28	180.0
C6—C5—C51	118.64 (16)	N28^i^—Ni2—N25^i^	91.69 (6)
C4—C5—C51	120.51 (16)	N28—Ni2—N25^i^	88.31 (6)
C5—C6—N22	126.42 (16)	N28^i^—Ni2—N25	88.31 (6)
C5—C6—C7	123.47 (16)	N28—Ni2—N25	91.69 (6)
N22—C6—C7	110.11 (15)	N25^i^—Ni2—N25	180.0
C8—C7—C6	107.54 (16)	C21—N25—C24	104.31 (15)
C8—C7—H7A	126.2	C21—N25—Ni2	126.14 (12)
C6—C7—H7A	126.2	C24—N25—Ni2	129.55 (12)
C7—C8—C9	106.44 (16)	C39—N28—C36	104.53 (14)
C7—C8—H8A	126.8	C39—N28—Ni2	126.32 (12)
C9—C8—H8A	126.8	C36—N28—Ni2	129.15 (12)
N22—C9—C10	125.99 (16)	N25—C21—C40	125.86 (16)
N22—C9—C8	111.37 (15)	N25—C21—C22	111.58 (16)
C10—C9—C8	122.63 (17)	C40—C21—C22	122.52 (17)
C9—C10—C11	123.98 (17)	C23—C22—C21	106.51 (16)
C9—C10—H10A	118.0	C23—C22—H22A	126.7
C11—C10—H10A	118.0	C21—C22—H22A	126.7
C10—C11—N23	125.79 (16)	C22—C23—C24	107.43 (16)
C10—C11—C12	122.87 (16)	C22—C23—H23A	126.3
N23—C11—C12	111.34 (15)	C24—C23—H23A	126.3
C13—C12—C11	106.63 (16)	C25—C24—N25	125.59 (16)
C13—C12—H12A	126.7	C25—C24—C23	124.25 (16)
C11—C12—H12A	126.7	N25—C24—C23	110.15 (15)
C12—C13—C14	107.52 (15)	C24—C25—C36^i^	120.86 (16)
C12—C13—H13A	126.2	C24—C25—C251	120.56 (16)
C14—C13—H13A	126.2	C36^i^—C25—C251	118.58 (16)
C15—C14—N23	125.46 (16)	N28—C36—C25^i^	126.47 (16)
C15—C14—C13	124.44 (16)	N28—C36—C37	110.24 (16)
N23—C14—C13	110.10 (15)	C25^i^—C36—C37	123.29 (16)
C16—C15—C14	121.16 (16)	C38—C37—C36	107.33 (16)
C16—C15—C151	118.16 (16)	C38—C37—H37A	126.3
C14—C15—C151	120.67 (16)	C36—C37—H37A	126.3
C15—C16—N24	126.37 (16)	C37—C38—C39	106.44 (16)
C15—C16—C17	123.36 (16)	C37—C38—H38A	126.8
N24—C16—C17	110.26 (15)	C39—C38—H38A	126.8
C18—C17—C16	107.38 (16)	N28—C39—C40	125.81 (16)
C18—C17—H17A	126.3	N28—C39—C38	111.44 (15)
C16—C17—H17A	126.3	C40—C39—C38	122.73 (17)
C17—C18—C19	106.45 (16)	C39—C40—C21	124.11 (17)
C17—C18—H18A	126.8	C39—C40—H40A	117.9
C19—C18—H18A	126.8	C21—C40—H40A	117.9
N24—C19—C20	125.87 (16)	C25—C251—C252	115.43 (14)
N24—C19—C18	111.62 (15)	C25—C251—H25B	108.4
C20—C19—C18	122.51 (17)	C252—C251—H25B	108.4
C1—C20—C19	123.85 (17)	C25—C251—H25C	108.4
C1—C20—H20A	118.1	C252—C251—H25C	108.4
C19—C20—H20A	118.1	H25B—C251—H25C	107.5
C5—C51—C52	116.05 (14)	C253—C252—C254	110.50 (15)
C5—C51—H51A	108.3	C253—C252—C251	108.62 (14)
C52—C51—H51A	108.3	C254—C252—C251	112.58 (15)
C5—C51—H51B	108.3	C253—C252—H25D	108.3
C52—C51—H51B	108.3	C254—C252—H25D	108.3
H51A—C51—H51B	107.4	C251—C252—H25D	108.3
C53—C52—C54	111.04 (15)	C252—C253—H25E	109.5
C53—C52—C51	108.59 (15)	C252—C253—H25F	109.5
C54—C52—C51	112.07 (15)	H25E—C253—H25F	109.5
C53—C52—H52A	108.3	C252—C253—H25G	109.5
C54—C52—H52A	108.3	H25E—C253—H25G	109.5
C51—C52—H52A	108.3	H25F—C253—H25G	109.5
C52—C53—H53A	109.5	C252—C254—H25H	109.5
C52—C53—H53B	109.5	C252—C254—H25I	109.5
H53A—C53—H53B	109.5	H25H—C254—H25I	109.5
C52—C53—H53C	109.5	C252—C254—H25J	109.5
H53A—C53—H53C	109.5	H25H—C254—H25J	109.5
H53B—C53—H53C	109.5	H25I—C254—H25J	109.5
			
N24—Ni1—N21—C1	2.07 (14)	C14—C15—C16—C17	−179.05 (15)
N22—Ni1—N21—C1	−179.29 (14)	C151—C15—C16—C17	2.0 (2)
N24—Ni1—N21—C4	−175.14 (14)	C19—N24—C16—C15	−178.49 (16)
N22—Ni1—N21—C4	3.50 (14)	Ni1—N24—C16—C15	2.6 (3)
N23—Ni1—N22—C9	−2.96 (14)	C19—N24—C16—C17	0.25 (18)
N21—Ni1—N22—C9	177.00 (14)	Ni1—N24—C16—C17	−178.67 (11)
N23—Ni1—N22—C6	176.73 (14)	C15—C16—C17—C18	178.55 (16)
N21—Ni1—N22—C6	−3.31 (14)	N24—C16—C17—C18	−0.2 (2)
N24—Ni1—N23—C11	−178.31 (14)	C16—C17—C18—C19	0.11 (19)
N22—Ni1—N23—C11	3.05 (14)	C16—N24—C19—C20	−179.83 (16)
N24—Ni1—N23—C14	0.39 (14)	Ni1—N24—C19—C20	−0.9 (2)
N22—Ni1—N23—C14	−178.25 (14)	C16—N24—C19—C18	−0.18 (18)
N23—Ni1—N24—C19	179.15 (14)	Ni1—N24—C19—C18	178.77 (11)
N21—Ni1—N24—C19	−0.81 (14)	C17—C18—C19—N24	0.0 (2)
N23—Ni1—N24—C16	−2.16 (14)	C17—C18—C19—C20	179.71 (16)
N21—Ni1—N24—C16	177.88 (14)	N21—C1—C20—C19	−0.4 (3)
C4—N21—C1—C20	175.90 (16)	C2—C1—C20—C19	176.13 (16)
Ni1—N21—C1—C20	−1.9 (2)	N24—C19—C20—C1	1.8 (3)
C4—N21—C1—C2	−0.93 (18)	C18—C19—C20—C1	−177.79 (16)
Ni1—N21—C1—C2	−178.70 (11)	C6—C5—C51—C52	−79.0 (2)
N21—C1—C2—C3	1.3 (2)	C4—C5—C51—C52	101.41 (19)
C20—C1—C2—C3	−175.68 (16)	C5—C51—C52—C53	176.87 (15)
C1—C2—C3—C4	−1.03 (19)	C5—C51—C52—C54	−60.1 (2)
C1—N21—C4—C5	−179.38 (16)	C16—C15—C151—C152	76.6 (2)
Ni1—N21—C4—C5	−1.7 (2)	C14—C15—C151—C152	−102.33 (19)
C1—N21—C4—C3	0.27 (18)	C15—C151—C152—C154	−175.11 (15)
Ni1—N21—C4—C3	177.95 (11)	C15—C151—C152—C153	61.2 (2)
C2—C3—C4—C5	−179.83 (16)	N28^i^—Ni2—N25—C21	−178.71 (14)
C2—C3—C4—N21	0.51 (19)	N28—Ni2—N25—C21	1.29 (14)
N21—C4—C5—C6	−1.8 (3)	N28^i^—Ni2—N25—C24	2.36 (14)
C3—C4—C5—C6	178.60 (16)	N28—Ni2—N25—C24	−177.64 (14)
N21—C4—C5—C51	177.76 (15)	N25^i^—Ni2—N28—C39	−178.68 (14)
C3—C4—C5—C51	−1.8 (3)	N25—Ni2—N28—C39	1.32 (14)
C4—C5—C6—N22	2.0 (3)	N25^i^—Ni2—N28—C36	1.21 (14)
C51—C5—C6—N22	−177.56 (15)	N25—Ni2—N28—C36	−178.79 (14)
C4—C5—C6—C7	−177.16 (16)	C24—N25—C21—C40	176.54 (16)
C51—C5—C6—C7	3.3 (2)	Ni2—N25—C21—C40	−2.6 (3)
C9—N22—C6—C5	−178.95 (16)	C24—N25—C21—C22	−1.09 (19)
Ni1—N22—C6—C5	1.3 (2)	Ni2—N25—C21—C22	179.76 (11)
C9—N22—C6—C7	0.32 (18)	N25—C21—C22—C23	1.4 (2)
Ni1—N22—C6—C7	−179.43 (11)	C40—C21—C22—C23	−176.30 (16)
C5—C6—C7—C8	178.30 (16)	C21—C22—C23—C24	−1.10 (19)
N22—C6—C7—C8	−0.99 (19)	C21—N25—C24—C25	179.50 (16)
C6—C7—C8—C9	1.20 (19)	Ni2—N25—C24—C25	−1.4 (2)
C6—N22—C9—C10	−178.37 (16)	C21—N25—C24—C23	0.38 (18)
Ni1—N22—C9—C10	1.4 (2)	Ni2—N25—C24—C23	179.49 (11)
C6—N22—C9—C8	0.44 (18)	C22—C23—C24—C25	−178.65 (16)
Ni1—N22—C9—C8	−179.81 (11)	C22—C23—C24—N25	0.5 (2)
C7—C8—C9—N22	−1.1 (2)	N25—C24—C25—C36^i^	−1.6 (3)
C7—C8—C9—C10	177.79 (16)	C23—C24—C25—C36^i^	177.35 (16)
N22—C9—C10—C11	1.4 (3)	N25—C24—C25—C251	178.48 (15)
C8—C9—C10—C11	−177.30 (16)	C23—C24—C25—C251	−2.5 (3)
C9—C10—C11—N23	−1.3 (3)	C39—N28—C36—C25^i^	−179.01 (16)
C9—C10—C11—C12	178.34 (16)	Ni2—N28—C36—C25^i^	1.1 (3)
C14—N23—C11—C10	179.45 (16)	C39—N28—C36—C37	0.88 (18)
Ni1—N23—C11—C10	−1.6 (2)	Ni2—N28—C36—C37	−179.03 (11)
C14—N23—C11—C12	−0.20 (18)	N28—C36—C37—C38	−0.1 (2)
Ni1—N23—C11—C12	178.77 (11)	C25^i^—C36—C37—C38	179.78 (16)
C10—C11—C12—C13	−179.53 (16)	C36—C37—C38—C39	−0.68 (19)
N23—C11—C12—C13	0.1 (2)	C36—N28—C39—C40	177.03 (16)
C11—C12—C13—C14	0.00 (19)	Ni2—N28—C39—C40	−3.1 (2)
C11—N23—C14—C15	−179.85 (16)	C36—N28—C39—C38	−1.32 (19)
Ni1—N23—C14—C15	1.2 (2)	Ni2—N28—C39—C38	178.59 (11)
C11—N23—C14—C13	0.20 (18)	C37—C38—C39—N28	1.3 (2)
Ni1—N23—C14—C13	−178.72 (11)	C37—C38—C39—C40	−177.12 (16)
C12—C13—C14—C15	179.92 (16)	N28—C39—C40—C21	2.0 (3)
C12—C13—C14—N23	−0.13 (19)	C38—C39—C40—C21	−179.87 (16)
N23—C14—C15—C16	−1.5 (3)	N25—C21—C40—C39	1.0 (3)
C13—C14—C15—C16	178.50 (16)	C22—C21—C40—C39	178.43 (16)
N23—C14—C15—C151	177.48 (15)	C24—C25—C251—C252	100.67 (19)
C13—C14—C15—C151	−2.6 (3)	C36^i^—C25—C251—C252	−79.2 (2)
C14—C15—C16—N24	−0.5 (3)	C25—C251—C252—C253	177.00 (15)
C151—C15—C16—N24	−179.42 (15)	C25—C251—C252—C254	−60.3 (2)

Symmetry code: (i) −*x*, −*y*+1, −*z*.

### Comparison of Ni^II^ 5,15-dialkylporphyrins (Å, °).

Δ is the deviation from the least-squares-plane of the 24 macrocycle atoms and N—Ni—Nadj is the angle between neighboring pyrrole units.

**Table:**

Alkyl residue	*tert*-Butyl	Isopropyl	*iso*-Butyl	none
Ni—N	1.897 (2)	1.930 (2)	1.955 (2)	1.951 (2)
Δ	0.4	0.26	0.04	0.02
N—Ni—N adj	92.3, 87.7	91.8, 88.2	91.6, 88.4	90, 90
Reference	Song *et al.* (1996)	Song *et al.* (1998)	This work	Jentzen *et al.* (1996)
